# Public opinion of the Irish “COVID Tracker” digital contact tracing App: A national survey

**DOI:** 10.1177/20552076221085065

**Published:** 2022-03-16

**Authors:** Michael E O’Callaghan, Manzar Abbas, Jim Buckley, Brian Fitzgerald, Kevin Johnson, John Laffey, Bairbre McNicholas, Bashar Nuseibeh, Derek O’Keeffe, Sarah Beecham, Abdul Razzaq, Kaavya Rekanar, Ita Richardson, Andrew Simpkin, James O’Connell, Cristiano Storni, Damyanka Tsvyatkova, Jane Walsh, Thomas Welsh, Liam G Glynn

**Affiliations:** 1 School of Medicine, 8808 University of Limerick, Limerick, Ireland; 2 Irish College of General Practitioners, Dublin, Ireland; 3 Lero - The Irish Software Research Centre, Tierney Building, 8808 University of Limerick, Ireland; 4 Department of Computer Science and Information Systems, 8808 University of Limerick, Ireland; 5 Department of Nursing and Midwifery, 8808 University of Limerick, Ireland; 6 School of Medicine, 8799 National University of Ireland Galway (NUIG), Galway, Ireland; 7 School of Mathematics, Statistics and Applied Mathematics, 8799 National University of Ireland Galway, Galway, Ireland; 8 School of Psychology, 8799 National University of Ireland Galway, Galway, Ireland; 9 HRB Primary Care Clinical Trials Network Ireland, Ireland

**Keywords:** COVID-19, contact tracing, mHealth, public health, software applications

## Abstract

**Objective:**

This study aims to gather public opinion on the Irish “COVID Tracker” digital contact tracing (DCT) App, with particular focus on App usage, usability, usefulness, technological issues encountered, and potential changes to the App.

**Methods:**

A 35-item online questionnaire was deployed for 10 days in October 2020, 3 months after the launch of the Irish DCT App.

**Results:**

A total of 2889 completed responses were recorded, with 2553 (88%) respondents currently using the App. Although four in five users felt the App is easy to download, is easy to use and looks professional, 615 users (22%) felt it had slowed down their phone, and 757 (28%) felt it had a negative effect on battery life. Seventy-nine percent of respondents reported the App's main function is to aid contact tracing. Inclusion of national COVID-19 trends is a useful ancillary function according to 87% of respondents, and there was an appetite for more granular local data. Overall, 1265 (44%) respondents believed the App is helping the national effort, while 1089 (38%) were unsure.

**Conclusions:**

DCT Apps may potentially augment traditional contact tracing methods. Despite some reports of negative effects on phone performance, just 7% of users who have tried the App have deleted it. Ancillary functionality, such as up-to-date regional COVID-19, may encourage DCT App use. This study describes general positivity toward the Irish COVID Tracker App among users but also highlights the need for transparency on effectiveness of App-enabled contact tracing and for study of non-users to better establish barriers to use.

## Introduction

Despite the availability of novel and effective vaccinations, controlling SARS-CoV-2 viral spread to reduce global disruption and strain on healthcare systems continues to rely heavily on traditional strengths of public health medicine.^1^ A key aspect of this work is “contact tracing,” which aims to rapidly identify the close contacts of infected people so that they can be advised to limit their social interactions, thereby reducing onward spread of the pathogen.^2^

Novel software applications (“Apps”) that can potentially simplify the laborious work of manual contact tracing are a tempting prospect, particularly given their ability to immediately identify contacts who are unknown to cases. Given this potential, many countries have designed, developed, and deployed Apps before their efficacy has been established.^3,4^

On the 7 July 2020, the Irish health service launched the “COVID Tracker” App, which uses the Google/Apple Exposure Notification (GAEN) Application Programming Interface^5,6^ to record users' close contacts using Bluetooth low energy technology. While the App's main function is to assist with contact tracing, it also features information about regional COVID-19 trends in Ireland and allows users to log if they have any symptoms that might be COVID-19.^7^ The COVID Tracker can be used by anyone over 16 years if their smartphone is less than 5 years old, and logs contacts where phones are within 2 meters of each other for at least 15 min. Previous research undertaken prior to the launch of the App showed that 82% of respondents stated they would be likely to download such an App.^8^

The App launch was well received, with over 1 million downloads in the first 48 h.^9^ Twelve months after the launch there were 1.3 million active users,^10^ although this figure has risen steadily in recent months. As of November 2021, there are 1.7 million active users in Ireland, which equates to 43% of the population aged 16 years or more.^11,12^ This recent increase is likely due to a useful update to the App which allows it to stores a QR-code and information relating to individuals’ COVID vaccination certificates (which can then be scanned or shown at venues, restaurants, bars etc.). There are approximately 3.0–3.4 million smartphones in use in Ireland^13–15^ and 75% are less than 5 years old.^15^ Thus 1.7 million active users represent 67–76% of total possible users.

The early stages of the App deployment have not been without issue, including resurfacing of concerns relating to the Google Play store,^16^ and a widely reported issue with Android software where phones with the App began consuming their battery rapidly and heating up in early August 2020.^17,18^ Although this problem was quickly contained, preliminary analyses suggest this resulted in a significant number of de-installations.^19^

However, the COVID Tracker App, built by the Irish company NearForm, has generally been considered a success. It is open-source software and since July 2020 has thus far has been used in nine jurisdictions across North America and Europe, providing digital contact tracing for 55 million people.^20^

Despite vaccination efforts gathering momentum worldwide, recurrent waves of virus activity^21^ and the consequent emergence of variants^22^ suggest that continued reliance on traditional public health measures, including contact tracing, is likely to remain central to responses for at least the medium term. The contribution which mobile-enabled contact tracing Apps can make, for this and for future infectious disease outbreaks, requires further study.

This study aims to gather the Irish general public's perceptions of the COVID Tracker App, including an analysis of known issues. Gaining insight into user attitudes and beliefs around the App's usability and usefulness, in addition to suggested changes, may be relevant for future iterations of this and other similar Apps.

## Methods

A 35-item online survey was designed and piloted by researchers at the University of Limerick Graduate Entry Medical School (GEMS), Science Foundation Ireland Research Centre for Software (Lero), and the National University of Ireland Galway (NUIG) School of Psychology and College of Medicine, Nursing and Health Sciences. Many questions in the survey used Likert scales and pre-populated responses to minimize fatigue and allow respondents to complete the survey in 10 to 15 min (see Appendix 1 for CHERRIES checklist). Although a majority of closed questions were used, many contained free text “Other” boxes, which granted respondents the opportunity to offer their own thoughts and suggestions (see Appendix 2 for the final survey instrument).

Topics covered included respondent characteristics, their experience with the App (if any) and their attitudes relating to its usability and usefulness. Finally, respondents were asked their opinions on App technological issues and suggested App changes.

Ethical approval for this project was granted by the University of Limerick Education and Health Sciences (EHS) ethics committee [study ID 2020_04_18_EHS (ER), approved 21 May 2020]. At the time of survey deployment, Ireland was experiencing a relatively low level of COVID-19 activity, with a 14-day national incidence of 101 cases per 100,000 citizens^23^ as compared to a 14-day national incidence of over 1500 cases per 100,000 citizens seen in January 2021.^24^

### Recruitment

The final survey was published online using Qualtrics XM survey software. Access to the survey was granted by participants clicking on a hypertext link, and only one response per device was permitted. Participants were invited to engage with the survey in three different ways. Firstly, the survey link was published on social media platforms (Twitter, Facebook, LinkedIN, and Instagram) by the study authors, many of whom were active on national and local media in the early stages of the pandemic. Secondly, the link was posted on the University of Limerick (UL) website and sent out to University emailing lists for students and staff from the University of Limerick and National University of Ireland, Galway email lists. Finally, in an attempt to broaden the reach of the survey, the link was sent out via the popular “WhatsApp” Messenger App. The WhatsApp message was sent to private “Group conversations” that the authors are members of, and encouraged recipients to both take the survey and forward on the link to family and friends. The survey link was published on Friday 2 October 2020 at 12 pm. The survey was live for ten days and the survey authors posted messages with a link to the online survey via their social media profiles during this period to encourage engagement.

### Statistical analysis

All analyses were carried out in R v3.6. Descriptive statistics are used to present demographics and attitudes of the respondents to App. Non-representativeness of the sample (compared to national age profile, female to male ratio, and educational attainment trends) precluded weighting and use of inferential statistics.

## Results

### Respondent characteristics

There were 2889 complete responses to the survey. Responses were received from all 26 counties in the Republic of Ireland, with most responses received from the major population centers of Dublin, Cork, Limerick, and Galway. Ninety-three percent of respondents were female (Table 1). Regarding highest educational attainment, 2590 (90%) of respondents reported having a third-level qualification. Median age of respondents was 35 years, with a mean age of 36 years. Sixty-eight percent of respondents were moderately or very worried about COVID-19 activity at time of survey and 16% of the sample were in high or very high risk groups.

**Table 1.** Respondent characteristics (*n* = 2889).

Gender			Education			Age range			COVID-19 related worry			Risk group		
Female	2697	(93%)	Primary/none	13	(0%)	18–29 years	829	(29%)	Very worried	1471	(11%)	Very high	68	(2%)
Male	191	(7%)	Secondary	253	(9%)	30–41 years	1311	(45%)	Moderately worried	1268	(57%)	High	397	(14%)
-	1	(0%)	Tertiary	2590	(90%)	42–53 years	496	(17%)	Not very worried	122	(26%)	Low/medium	2347	(81%)
			-	33	(1%)	54–65 years	218	(8%)	Not at all worried	22	(6%)	I'm not sure	71	(2%)
						66 years plus	28	(1%)	I'm not sure	6	(0%)	-	6	(0%)
						-	7	(0%)						
Total	2889			2889			2889			2889			2889	

### App usage

Of the 2889 respondents, 2741 (95%) had downloaded the App at some point, while 2553 (88%) were still using it (Table 2). For the 188 (7%) respondents who had stopped using the App, 116 (62% of this group) reported battery-related concerns. For the 148 respondents (5%) who had never downloaded the App, 49 reported a privacy concern, 32 did not trust the Irish health service, 26 do not trust major technology companies, and 23 felt the App simply was not useful.

**Table 2.** Respondent App usage.

Downloaded App (ever)	2741	(95%)	Never Downloaded App	148	(5%)
Stopped using the App	188	(7%)	Privacy concern	49	(2%)
Stopped using the App due to Battery concerns	116	(4%)	Health service distrust	32	(1%)
Current Users	2553	(88%)	Technology company distrust	26	(1%)
			App not useful	23	(1%)

### App usability

For the 2741 participants who had downloaded the App at some point (described hereafter as “users”), 2095 (76% of users) strongly agreed that it was easy to download and set up, while 1845 (67% of users) strongly agreed the App was easy to use (Table 3). Just 123 (4%) respondents disagreed with the statement that the App looks professional and of high quality. Of the user group, 615 (22%) felt the App had at least somewhat slowed down their phone's performance and 757 (28%) reported it had some negative effect on their battery life.

**Table 3.** Respondent opinions on App usability/performance.

“Please select the option below that best describes how you feel about the following statements:”	Strongly agree		Somewhat agree		Neutral		Somewhat disagree			Strongly disagree		Skip			Users
The App was easy to download and get started	2095	(76%)	318	(12%)	45	(2%)		18	(1%)	84	(3%)	181	(7%)	2741	
The App is easy to use	1845	(67%)	529	(19%)	89	(3%)		30	(1%)	67	(2%)	181	(7%)	2741	
The App looks professional and of high quality	1445	(53%)	805	(29%)	187	(7%)		64	(2%)	59	(2%)	181	(7%)	2741	
The App has slowed down my phone's performance	150	(5%)	465	(17%)	750	(27%)		352	(13%)	843	(31%)	181	(7%)	2741	
The App causes my phone's battery to deplete quickly	250	(9%)	507	(18%)	655	(24%)		377	(14%)	771	(28%)	181	(7%)	2741	

### App functions

Respondents were asked about each of the App's three functions. Regarding the primary function of the App, 2333 (81%) of respondents feel this is clear. Seventy-nine percent of respondents felt the App's main function is to assist contact tracing efforts. The App also features regularly updated national and regional COVID-19 data, which 2504 (87%) respondents believe to be useful. Regarding the third and final function of the App, the Check-in feature where users log if they feel unwell, 1502 (52%) felt this information was likely to be of use to the Irish health service (Table 4).

**Table 4.** Responses relating to App functionality.

Is the main function of the HSE COVID Tracker App clear?			So what do you feel the MAIN function of the App is?			Do you think it is useful to have the latest Irish COVID-19 data available on the App?			Do you think it is useful to be able to tell the App/the HSE if you are feeling unwell?		
Yes	2333	(81%)	To help with Contact Tracing	2293	(79%)	Yes	2504	(87%)	Yes	1502	(52%)
I'm not sure	204	(7%)	The main function of the App is not clear	352	(12%)	I'm not sure	107	(4%)	I'm not sure	661	(23%)
No	352	(12%)	To give people information on COVID-19 trends	154	(5%)	No	62	(2%)	No	495	(17%)
			To allow people to upload symptoms	67	(2%)	Skip	216	(7%)	Skip	231	(8%)
			I'm not sure, skip this question	23	(1%)						
Total	2889		Total	2889		Total	2889		Total	2889	

### App usefulness

Overall 1265 (44%) respondents stated they believe the App is helping the national effort, while 1089 (38%) were unsure (Table 5). The remaining 535 (19%) respondents felt the App is not helping.

**Table 5.** Respondent demographics, COVID-related worry, and self-reported risk versus opinion on whether the App is helping the national response to COVID-19.

		“In your opinion, is the COVID Tracker App helping our national response to COVID-19?”						
		Yes		I'm not sure			No	
	Total	1265	(44%)	1089	(38%)	535		(19%)
Gender	Female	1154	(43%)	1029	(38%)	514		(19%)
	Male	111	(58%)	59	(31%)	21		(11%)
	Prefer not to say	0	-	1	(100%)	0		-
Age range	18–29 years	311	(38%)	304	(37%)	214		(26%)
	30–41 years	565	(43%)	509	(39%)	237		(18%)
	42–53 years	242	(49%)	188	(38%)	66		(13%)
	54–65 years	130	(60%)	75	(34%)	13		(6%)
	66 years plus	15	(54%)	11	(39%)	2		(7%)
	Undisclosed	2	(29%)	2	(29%)	3		(43%)
COVID-19-related worry	Very worried	684	(46%)	525	(36%)	262		(18%)
	Moderately worried	543	(43%)	517	(41%)	208		(16%)
	Not very worried	32	(26%)	39	(32%)	51		(42%)
	Not at all worried	4	(18%)	6	(27%)	12		(55%)
	I'm not sure	2	(33%)	2	(33%)	2		(33%)
Risk group	Very high	32	(47%)	23	(34%)	13		(19%)
	High	201	(51%)	139	(35%)	57		(14%)
	Low or medium	1008	(43%)	888	(38%)	451		(19%)
	I'm not sure	22	(31%)	39	(55%)	10		(14%)
	Undisclosed	2	(33%)	0	-	4		(67%)

Male respondents, though far fewer in number, were more positive about the App's contribution to the national effort (111/191 (58%) males vs. 1154/2697 (43%) females). Being in a low or medium risk group was associated with lower perception of usefulness, with 43% of these respondents feeling the App is helping the national effort compared to 50% of respondents in the high or very high risk group. Low levels of COVID-19-related worry were also associated with less positive views of the App's usefulness, with 44% of those “Not Very Worried” or “Not At All Worried” about COVID-19 reporting that they felt the App is not helping the national effort compared to 17% of those “Very Worried” or “Moderately Worried.”

Younger respondents were more negative about the App's usefulness, with 21% of those aged 41 years or younger reporting that it is not helping the national effort, compared to 11% of those 42 years or older.

### App issues

All respondents were asked about three well-publicized issues with the App since launched (Table 6). Sixty percent were aware that the App will not work with phones that are more than 5 years old, while 39% were aware of an Android software issue that meant some users were required to grant access the App access to “Location Services,” despite the App not accessing location data. Finally, 63% of respondents were aware of a software issue that caused some Android phones’ batteries to drain and to heat up considerably over a period of several days in August 2020. Of these issues, the battery drain was the most damaging issue examined, leading to 406 people in our sample to delete the App. However, 306 of this group only deleted the App temporarily, as a software update quickly fixed the issue.

**Table 6.** Awareness of and responses to technological issues since App launch.

Issue 1: Older phones			Issue 2: Google location services			Issue 3: Battery drain		
I was aware of this issue	1728	(60%)	I was aware of this issue	1124	(39%)	I was aware of this issue	1823	(63%)
I was not aware of this issue	1161	(40%)	I was not aware of this issue	1765	(61%)	I was not aware of this issue	1066	(37%)
Total	2889		Total	2889		Total	2889	
Issue 1: Response			Issue 2: Response			Issue 3: Response		
I downloaded the App	1180	(68%)	I downloaded the App	800	(71%)	I kept the App on my phone	1050	(58%)
Not applicable (skip)	510	(30%)	I deleted the App (temporarily)	36	(3%)	I deleted the App (temporarily)	306	(17%)
I did not download the App	38	(2%)	I deleted the App (permanently)	24	(2%)	I deleted the App (permanently)	100	(5%)
Subtotal	1728		I did not download the App	15	(1%)	I did not download the App	38	(2%)
			Not applicable (skip)	249	(22%)	Not applicable (skip)	329	(18%)
			Subtotal	1124		Subtotal	1823	

### App modifications

When presented with a list of potential changes to the App, 2284 (79%) of our sample would like to see more COVID-19 trend data relating to their local area (Table 7). Many respondents also indicated they would share more personal information with the App if it was shown to help public health teams, including when they were tested recently (76%), their age (63%), their location (58%), and their ethnicity (49%).

**Table 7.** Respondent opinions on changes to how a) the App presents information and b) users might be able to enter more information into the App.

a) “Are there any changes to how the App presents information you would like to see?”			b) “Are there any changes to how you enter data into the App that you would like to see implemented?”		
I would like the App to have more information about COVID-19 trends in my local area	2284	(79%)	I would allow the App to know if I had been tested for COVID-19 recently if it helped public health teams	2207	(76%)
I would like the App to tell me how many other people with the App I've been in close contact with recently	1598	(55%)	I would allow the App to know my Age if it helped public health teams	1830	(63%)
I would like the App to give more up-to-date information on COVID-19 in Ireland	1179	(41%)	I would allow the App to know my recent Location data if it helped public health teams	1674	(58%)
No I don't think these ideas/changes would be useful	99	(3%)	I would allow the App to know my Ethnicity if it helped public health teams	1418	(49%)
			No I don't think these ideas/changes would be useful	198	(7%)

## Discussion

### Principal results

This online survey collected opinions about the Irish contact tracing App 3 months after its launch. The vast majority of respondents were current App users, and attitudes toward the App from a usability perspective were quite positive. Although approximately one quarter of users felt that the App had a negative effect on their phone's performance, just 7% of those who have downloaded and used the App are no longer using it. Thus, it seems that people are willing to tolerate a certain amount of inconvenience for the greater good. Although non-users of the App were underrepresented in our sample, those surveyed reported privacy and trust concerns as the principal barriers to App adoption, which is in keeping with previous research.^8^

Regarding the various functions of the App, almost 9 in 10 respondents reported that the COVID-19 data contained in the App are useful, and indeed 4 in 5 would like to have more granular data available to inform them if COVID-19 is spreading locally. However, 21% of respondents were not clear that the App's main function is to aid contact tracing. Additionally, just over half of respondents felt the check-in function was likely to be of use to the health service, indicating considerable uncertainty among many users around some aspects of the App's functionality.

Indeed, just under half of our respondents felt that the COVID Tracker App is helping our national effort. Official communications around the overall effectiveness of the App, particularly around how much it is helping contact tracing efforts, but also including such features as the check-in function, are lacking.

There is also a dearth of information available online from official sources regarding the technological issues examined in this study. Nevertheless, a majority of respondents were aware of the widely reported battery-drain issue which caused phones to heat up in August 2020, presumably via mainstream media.^17,18^ The battery drain issue was the most damaging of the issues examined herein, although re-installation of the App in three-quarters of cases demonstrates that many users were reassured by the quick response to address this issue.

Feedback from the general public about suggested App changes seems to indicate, in our respondent group at least, a willingness to share more personal information if it is proven to assist public health efforts, which seems to be a rational and reassuring response during this time of crisis. There was also strong support for expanding the regional COVID-19 data function of the App to include more granular data at a local level.

These findings may support App developers and health authorities enhancing communications around the contact tracing function of DCT Apps and being transparent as to their effectiveness. If these Apps are important public health tools, then communications around their operation should be based on the same models as traditional public health advice, and should come from official sources. If these Apps are not useful in their current form, they clearly need refinement.

### DCT App effectiveness

This study has touched on the fact that it is difficult to ascertain the benefit of DCT Apps with privacy by design features deployed in the midst of a health emergency. However, we know that these Apps can only be effective if the technology is sound and if they are adopted by large numbers of people.

Concerns expressed about the use of Bluetooth low energy technology accuracy and strict application of 2-meter distance rules warrant further investigation also, particularly as our understanding of how this novel virus spreads evolves.^25,26^ Further study is also required to disentangle appraisal of GAEN-based contact tracing Apps versus QR-code based functionality, which are used by some countries’ DCT Apps to log entry of users to a given location or venue.^27^

Real-world testing of GAEN-based contact tracing Apps has shown they can be more successful than traditional contact tracing methods, presumably due to their ability to identify unknown contacts.^7,28,29^ Nevertheless, it is well recognized that more formal assessments of DCT Apps are now required.^30^

In terms of adoption, the other critical factor for success, the Irish COVID Tracker App adoption swiftly plateaued, which has been the experience of many similar democracies.^31–35^ The importance of having widespread use of DCT Apps is underlined by the simple formula X^2^, with increasing gains in contacts recorded as the proportion of App users in the population (X) rises.

Interestingly, in recent months, active users of the Irish DCT App have increased by a further 30%. We can only assume is as a result of an additional new function that allows users to scan an official COVID-19 vaccination certificate into their local version of the App, thereby giving them a convenient official “COVID-pass” for use when entering venues, restaurants, etc.

As of 11 November 2021, 20,563 users of the COVID Tracker App who have tested positive for COVID-19 have uploaded a code given to them by public health authorities to their phone's App. This represents 4.3% of those newly infected over the 16-month period since the App launch.^11,36^ In doing so they have notified 33,839 other App users of recent close contact with someone who has tested positive for COVID-19.^11^ How many of these 33,839 people were already notified directly by their close contact, or indeed by public health authorities, prior to getting the App notification is unclear. Additionally, how these additional warnings in 4.3% of infections impact on behavior and subsequent transmission is unclear given available data. Therefore, it is impossible to determine how effective the App has been at assisting the contact tracing process, and thus reducing viral spread, with the information available to date for Ireland.

However, given what is at stake, additional work by public health authorities to clearly delineate cases where Apps have offered more timely warning to potentially infected individuals, perhaps through additional questioning from human contact tracers, should be prioritized. Indeed, if these Apps are proven to be of use to the public health response, gathering opinions from a sample of the remaining 800,000 people in Ireland who could have downloaded, but have as yet avoided, the App would be valuable.

Thus, our study adds to the growing literature on faltering adoption rates and technological challenges associated with the appraisal of COVID-19 contact tracing Apps.^25,28,29,32,34,37^ However, emerging evidence from other countries holds some promise. From the UK, a recently published study estimates several hundred thousand infections were likely prevented by the widely adopted GAEN-based NHS COVID-19 App.^38^

## Limitations

Our survey yielded a sample where 88% were using the App, which is clearly at odds with the 43% of active users nationally. Thus, it is likely that the survey appealed to those willing to adopt the App and understanding more about barriers to adoption requires further study. However, our survey sought to gather opinions on the functionality of the App and its perceived contribution to the national COVID-19 response, questions those with direct experience of the App may be best placed to answer.

Respondents were asked several open questions to identify concerns they may have about the App (see Appendix 2) and no prevalent concerns emerged that might have been anticipated from previous research,^8^ which suggests that there were no major “unknown reasons” identified by this survey that might be reducing interaction with the App by the general public.

Although selection bias and other sampling issues inherent in online surveys are well described,^39–42^ this survey had a large sample size and reached a geographically dispersed group of respondents. Nevertheless, our sample was not representative in terms of educational attainment, gender, and age.

Those with higher educational attainment were most likely overrepresented in the sample due to the use of University emailing lists and the social media profiles of University academic staff to encourage completion of the survey. The use of WhatsApp messenger to distribute the survey link among family and friends was an attempt to reduce this source of bias, but it clearly was not as effective as we had hoped.

The sample was also very heavily skewed toward female participants. Although many studies have indicated females are more likely to respond to online surveys,^39,40^ the extent of our disproportionate representation of females is largely explained by our online survey invitation gaining traction with the online “followers” of a popular female Irish medical doctor who encouraged completion of the survey on two separate days during the survey window. These endorsements were followed by a noticeable spike in engagement from young female respondents, with 75% of overall responses to our survey being gathered in the 24-h period after each post

Our data also contain an overrepresentation of younger age groups, which can be a further limitation of online surveys.^40–42^ However, we know that young people are more likely to mix socially^43^ and those less than 45 years of age comprise more than 67% of confirmed cases to date.^36^ Thus, gathering their views on the App is important. In addition, we have shown these groups are also the least convinced regarding the App's main function and its usefulness. These findings highlight a need to target younger age groups if greater App adoption proves to be a worthwhile endeavor.

## Conclusions

Contact tracing Apps have the potential to augment traditional contact tracing methods but can only be effective if the technology is sound and they are widely adopted. Understanding user experience and attitudes regarding DCT Apps may assist in improving App adoption.

More than 40% of the public in Ireland are currently using the Irish DCT App and respondents in this study indicated a willingness to put up with a certain amount of inconvenience for the greater good, as well as an understandable desire to get more granular information about the situation “close to home.” Official communications around the main function of the App as a tool to assist contact tracing may need bolstering. Also, it is reasonable to suggest that transparency and clear metrics relating to App close-contact notifications from official sources are also needed. Objective evidence of the impact these Apps are having could be used in communications designed to improve their further adoption and consequent usefulness.

If GAEN-based DCT Apps are determined to be ineffective as contact tracing tools, this knowledge would encourage refinement and improvement of this technology.

## Supplemental Material

sj-docx-1-dhj-10.1177_20552076221085065 - Supplemental material for Public opinion of the Irish “COVID Tracker” digital contact tracing App: A national survey Click here for additional data file. Supplemental material, sj-docx-1-dhj-10.1177_20552076221085065 for Public opinion of the Irish “COVID Tracker” digital contact tracing App: A national survey by Michael E O’Callaghan, Manzar Abbas, Jim Buckley, Brian Fitzgerald, Kevin Johnson, John Laffey, Bairbre McNicholas, Bashar Nuseibeh, Derek O’Keeffe, Sarah Beecham, Abdul Razzaq, Kaavya Rekanar, Ita Richardson, Andrew Simpkin, James O’Connell, Cristiano Storni, Damyanka Tsvyatkova, Jane Walsh, Thomas Welsh and Liam G Glynn in Digital Health

sj-docx-2-dhj-10.1177_20552076221085065 - Supplemental material for Public opinion of the Irish “COVID Tracker” digital contact tracing App: A national survey Click here for additional data file. Supplemental material, sj-docx-2-dhj-10.1177_20552076221085065 for Public opinion of the Irish “COVID Tracker” digital contact tracing App: A national survey by Michael E O’Callaghan, Manzar Abbas, Jim Buckley, Brian Fitzgerald, Kevin Johnson, John Laffey, Bairbre McNicholas, Bashar Nuseibeh, Derek O’Keeffe, Sarah Beecham, Abdul Razzaq, Kaavya Rekanar, Ita Richardson, Andrew Simpkin, James O’Connell, Cristiano Storni, Damyanka Tsvyatkova, Jane Walsh, Thomas Welsh and Liam G Glynn in Digital Health
