# Supplementary material for: Public opinion of the Irish “COVID Tracker” digital contact tracing App: A national survey
Source: Digit Health. 2022 Mar 16;8:20552076221085065. doi: 10.1177/20552076221085065 (PMC8935577; doi:10.1177/20552076221085065)
Supplement: sj-docx-1-dhj-10.1177_20552076221085065 - Supplemental material for Public opinion of the Irish “COVID Tracker” digital contact tracing App: A national survey [file sj-docx-1-dhj-10.1177_20552076221085065.docx]

## Appendix 1

| **Checklist for Reporting Results of Internet E-Surveys (CHERRIES)**  Eysenbach G. Improving the quality of Web surveys: the Checklist for Reporting Results of Internet E-Surveys (CHERRIES). J Med Internet Res. 2004 Sep 29;6(3):e34. doi: 10.2196/jmir.6.3.e34. Erratum in: doi:10.2196/jmir.2042. | | |
| --- | --- | --- |
| **Item Category** | **Checklist Item** | **Explanation** |
| Design | Describe survey design | A convenience sample of those using popular social media platforms (Twitter, Facebook), the WhatsApp messenger platform and university mailing lists (University of Limerick and National University of Ireland, Galway). |
| IRB (Institutional Review Board) approval and informed consent process | IRB approval | Approved by University of Limerick Education and Health Sciences Ethics committee on 21st May 2020 [study ID 2020_04_18_EHS ER]. |
|  | Informed consent | Following potential participants clicking on a link sent out via the above methods, they were brought to an initial information page. This told them the length of time of the survey, who the investigators were, the purpose of the study and the anonymised nature of the survey. |
|  | Data protection | No identifiable information was collected by the researchers. |
| Development and pre-testing | Development and testing | The survey was developed based on a previous survey deployed by this research group earlier in the pandemic. Specific questions on digital literacy were taken from the eHealth Literacy Assessment Toolkit - eHLA [Karnoe A, Furstrand D, Christensen K, Norgaard O, Kayser L. Assessing Competencies Needed to Engage With Digital Health Services: Development of the eHealth Literacy Assessment Toolkit. J Med Internet Res 2018;20(5):e178] |
| Recruitment process and description of the sample having access to the questionnaire | Open survey versus closed survey | Open survey accessed by clicking a unique link to take you launch page of survey delivered via Qualtrics platform. |
|  | Contact mode | Initial contact with the potential participants was made on the Internet (social media sites Twitter and Facebook), via email or via the messaging App "WhatsApp". |
|  | Advertising the survey | Online- via Twitter, WhatsApp, Facebook, University emailing lists (University of Limerick and National University of Ireland, Galway) and on the University of Limerick COVID-19 information page. Twitter:- Can technology help us in the fight against COVID-19? Please muster the energy (+ 10 mins) to take yet another survey (link below) on the COVID Tracker App... 4/4 *** retweets appreciated *** ['survey link'] WhatsApp:- Morning all, I know survey fatigue might be setting in but we would really appreciate if ye took this survey on the COVID Tracker App + forwarded it on. "Have your say on next steps for the 🇮🇪 COVID Tracker App! Please consider taking our 10 min survey via the link below...  ['survey link'] Many thanks, Dr Mike O Callaghan, Prof Liam Glynn. GPs, UL School of Medicine" |
| Survey administration | Web/E-mail | Our e-survey was delivered via the Qualtrics platform. Participants needed to click on a link to begin the survey. |
|  | Context | Online- via Twitter, WhatsApp, Facebook, University emailing lists (University of Limerick and National University of Ireland, Galway) and on the University of Limerick COVID-19 information page. |
|  | Mandatory/voluntary | This was a voluntary survey. |
|  | Incentives | No incentives involved. |
|  | Time/Date | 10 day period from Friday 2nd October (midday) to Monday 11th October (midday) |
|  | Randomization of items or questionnaires | No randomisation of survey questions was employed. |
|  | Adaptive questioning | Adaptive questioning (certain items, or only conditionally displayed based on responses to other items) were used to reduce number and complexity of the questions. |
|  | Number of Items | In general, 1 to 2 items were asked per page. For questions with some pre-text to read, generally only 1 question was asked, but for simpler questions, 2 questions were presented to the user per page. The total number of possible items was 35 questions, though many users would not be taken to each question based on their responses (i.e. adaptive questioning as outlined above). |
|  | Number of screens (pages) | The average user needed to submit answers across 25 screens to complete the survey. |
|  | Completeness check | Using Qualtrics inbuilt programming, checks for completeness were done at time of data entry. Data validation of some answers was also employed (e.g. ensuring Age is a whole number). All items provided a non-response option such as “not applicable” or “I'm not sure - skip” option. Items where multiple options could be selected were clearly flagged as such. |
|  | Review step | Once an answer was submitted (and met the data validation rules used for some questions), it could not be changed. |
| Response rates | Unique site visitor | View rates were not possible as users started the survey by clicking on a link distributed across many platforms. The in-built Qualtrics option to use IP addresses to prevent 2 responses from the same device was used, although these were not collected by the researchers (Qualtrics instead provides a unique code for each respondent). |
|  | View rate (Ratio of unique survey visitors/unique site visitors) | View rates were not possible as users started the survey by clicking on a link distributed across many platforms. The in-built Qualtrics option to use IP addresses to prevent 2 responses from the same device was used, although these were not collected by the researchers (Qualtrics instead provides a unique code for each respondent). |
|  | Participation rate (Ratio of unique visitors who agreed to participate/unique first survey page visitors) | Participation rates were not possible as accurate figures for the denominator (unique first page completions) cannot be calculated from information available. |
|  | Completion rate (Ratio of users who finished the survey/users who agreed to participate) | It is possible through the Qualtrics output to determine that in addition to the 2,889 complete responses, there were 271 partial responses (8.6% of 3,160 people who filled out the first survey page (i.e. the consent form)). The 271 partial responses were on average 46% complete, and these users spent median 3mins 54secs completing the survey. Of note, the survey was closed after 10 days and after this point users could no longer continue to fill in the survey. |
| Preventing multiple entries from the same individual | Cookies used | N/A |
|  | IP check | The in-built Qualtrics option to use IP addresses to prevent 2 responses from the same device was used, although these were not collected by the researchers (Qualtrics instead provides a unique code for each respondent). |
|  | Log file analysis | N/A |
|  | Registration | N/A |
| Analysis | Handling of incomplete questionnaires | Only completed questionnaires were analysed. |
|  | Questionnaires submitted with an atypical timestamp | N/A |
|  | Statistical correction | Statistical testing carried out by our team deemed weighting or adjustment were not appropriate for our non-representative sample. Thus results as reported are the raw data recorded. |
