# Supplementary material for: Public opinion of the Irish “COVID Tracker” digital contact tracing App: A national survey
Source: Digit Health. 2022 Mar 16;8:20552076221085065. doi: 10.1177/20552076221085065 (PMC8935577; doi:10.1177/20552076221085065)
Supplement: sj-docx-2-dhj-10.1177_20552076221085065 - Supplemental material for Public opinion of the Irish “COVID Tracker” digital contact tracing App: A national survey [file sj-docx-2-dhj-10.1177_20552076221085065.docx]

## Appendix 2

Q1 Thank you for considering taking our survey on COVID-19 and the HSE COVID Tracker App... **Dr Michael O’Callaghan** GP + Researcher                    **Prof Liam Glynn** GP + Professor of General Practice University of Limerick, Ireland
  
Before you take the survey, please read the following:
 **Study Title:** “Impact of COVID-19 in Ireland and the role of technology (Part 2)” **Principal Investigator:** Dr Michael O’Callaghan (University of Limerick) **Background information:** The SARS-CoV-2 Coronavirus (which causes the disease COVID-19) was declared a global health emergency by the World Health Organisation on 11th March 2020. An important part of limiting transmission of SARS-CoV-2 involves “contact tracing”, where people who catch the virus are asked who they have been in contact with recently. Contact tracing helps to find any people who might be spreading the virus in our communities, whether they feel ill or not. Once identified, we can warn these people to stay away from others for 2 weeks and this then helps to prevent further cases of COVID-19. On 22/05/20 our research team deployed a survey to ask the Irish general public what they felt about a new software App to help in Ireland’s contact tracing efforts. We received more than 8,000 replies, thank you to all who responded. **What is the reason for this second survey?:** The Health Service Executive (HSE) released Ireland’s COVID Tracker App on 07/07/20 which has 3 functions: Digital contact tracing of close contacts of confirmed cases of COVID-19 Allows users to record if they have symptoms Provide daily information about COVID-19 from a trusted source This second survey aims to understand more about the experience of the Irish general public of the COVID Tracker App and suggestions they might have for its improvement. **Why am I being asked to take part?:** While Ireland’s COVID Tracker has been downloaded more than 1.7 million times, it has had a few issues since its launch. We know that people will need to trust and use these types of Apps on an ongoing basis if they are to be helpful to our COVID-19 effort. Therefore it is important we try and gather as much information as possible on issues people might be having with the App, in addition to suggestions for improvements. **What will you have to do?:** We are asking you complete an anonymous survey. Answering these questions should take approximately 10 minutes of your time. If you do decide to take the survey, you may withdraw at any time for any reason by just closing your Internet browser. **What are the benefits?:** Your answers will help us understand more about how the Irish public view the HSE’s COVID Tracker App and if there are things that need to be improved if this software tool is going to help our ongoing response to COVID-19. **What are the risks?:** You might decide that you don’t want to answer a question. If this happens, most questions feature a “I'm not sure” option that will skip to the next question. **What happens to the information?:** Your answers will be completely anonymous. These answers will be stored in a password-protected file on a University of Limerick password-protected computer for the duration of this study. The answers we collect may be summarised and used in academic publications and in online reports describing the results of this survey. **What if you have more questions?:** If you would like to ask us any questions before deciding to take part, please contact the research team using the email address mike.ocallaghan@ul.ie     **Participant Consent**   Should you agree to participate in this study please read the statements below and if you agree to them, please tick the box below:   • I have read and understood the participant information above. • I confirm that I am aged 18 years or over and live on the island of Ireland. • I am fully aware of what I will have to do, and of any risks and benefits of the study. • I understand what the project is about, and what the results will be used for. • I understand that what the researchers find out in this study may be shared with others but that all answers I give will be anonymous. • I know that I am choosing to take part in the study and that I can stop taking part in the study at any stage without giving any reason by closing this browser window.

- I agree to the statements above and I consent to taking part in this research study (1)

Q1 What is your age?

________________________________________________________________

Q2 What is your Gender? (please select option that best applies)

- Male (1)
- Female (2)
- Other (3)
- Prefer not to say (4)

Q3 What is your highest level of educational attainment? (if a student, please indicate what qualification you hope to achieve)

- No formal education or training (1)
- Finished primary school (4)
- Finished secondary school (5)
- Skilled trade (6)
- 3rd level qualification (e.g. level 6 higher certificate or level 7/8 degree) (7)
- Post-grad 3rd level qualification (e.g. post-grad diploma, masters or PhD) (10)
- Prefer not to say (8)

Q4 In which county do you live?

▼ Antrim (1) ... Outside Ireland (33)

Q5 **COVID-19**   *Different people are determined to be at higher risk if they contract COVID-19. The categories used in Ireland are:* *Very High Risk: People aged over 70yrs, people on chemotherapy, people with immune system problems or severe lung conditions.* *High Risk: People aged over 60yrs, people with lung conditions or heart related conditions (e.g. high blood pressure) and people who are obese.* *Medium or Low Risk: People younger than 60yrs who don't suffer from a chronic illness or obesity are generally considered to be low or medium risk from COVID-19, as their chance of developing severe complications is much lower.*

 *What category below best describes you when it comes to COVID-19?*

- I'm in a Very High Risk group (1)
- I'm in a High Risk group (2)
- I'm in a Low or Medium Risk group (3)
- I'm not sure (4)
- Prefer not to say (skip) (5)

Q6 Is there someone else in your household, or who you have caring responsibilities for, in a high/very high risk group if they were ill with COVID-19?

- Not applicable, I don't live with or care for another person (6)
- A person I live with/care for is in a Very High Risk group (1)
- A person I live with/care for is in a High Risk group (2)
- People I live with/care for are in Low or Medium Risk groups (3)
- I'm not sure (4)
- Prefer not to say (skip) (5)

Q7 The 2019 coronavirus, otherwise known as COVID-19, is an infectious disease first identified in the city of Wuhan, China. Infections have since been reported around the world. Symptoms include fever, coughing and breathing difficulties.   How worried, if at all, are you about current coronavirus (i.e. COVID-19) activity in Ireland?

- Very Worried (1)
- Moderately Worried (2)
- Not Very Worried (3)
- Not At All Worried (4)
- I'm not sure (5)

*Display This Question:*

*If Q7 = Very Worried*

*Or Q7 = Moderately Worried*

Q8a Why are you worried about COVID-19 activity in Ireland? (please select any/all that apply)

- I worry a lot more people will die from COVID-19 if the virus spreads further (4)
- I worry a lot more people will die from other medical problems if the virus spreads further (5)
- I worry about the effects on peoples' mental health (6)
- I worry about the effects on jobs & the economy (7)
- I worry that not enough people are following public health advice (10)
- Other (8) ________________________________________________
- ⊗I'm not sure, skip this question (9)

*Display This Question:*

*If Q7 = Not Very Worried*

*Or Q7 = Not At All Worried*

Q8b Why are you not worried about COVID-19 activity in Ireland? (please select any/all that apply)

- I feel I am able to manage my risk by carefully following public health advice (10)
- I think the dangers associated with COVID-19 are overstated (4)
- I think our healthcare system has more important problems to deal with (5)
- I think our economy & peoples' jobs are more important at this stage (6)
- Other (9) ________________________________________________
- ⊗I'm not sure, skip this question (8)

Q9 **Technology**

 How familiar are these technology related terms to you?
  Please rate on a scale from 'Not at all familiar' to 'Completely familiar'.

|  | Not at all familiar (1) | Not very familiar (7) | Quite familiar (8) | Completely familiar (9) |
| --- | --- | --- | --- | --- |
| **Keyboard** (1) |  |  |  |  |
| **Settings** (2) |  |  |  |  |
| **Operating system (e.g. Windows)** (3) |  |  |  |  |
| **User name** (4) |  |  |  |  |

Q10
On a scale from 'Not at all confident' to 'Completely confident', please rate your use of computers.
 
How confident do you feel...

|  | Not at all confident (1) | Not very confident (2) | Quite confident (3) | Completely confident (4) |
| --- | --- | --- | --- | --- |
| **... using a computer in general?** (1) |  |  |  |  |
| **... using Touchscreen?** (2) |  |  |  |  |
| **... finding information online?** (3) |  |  |  |  |

Q11 Do you own a smartphone?

- Yes (1)
- No (2)
- I'm not sure (3)

*Display This Question:*

*If Q11 = Yes*

Q12 How long have you owned your current phone?

- Less than 6 months (1)
- Around 1 year (2)
- Around 2 years (3)
- Around 3 years (4)
- 4 or more years (5)

*Display This Question:*

*If Q11 != No*

Q13 **The HSE COVID Tracker App** On the 7th of July, the HSE released the COVID Tracker App.   Have you downloaded this App? (Note we'll ask in a moment if you still have the App on your phone)

- Yes (1)
- Yes, but it wouldn't work on my phone (2)
- No (4)

*Display This Question:*

*If Q13 = Yes, but it wouldn't work on my phone*

Q13a Why did the App not work on your phone?

- I have an older model (4)
- I don't know (5)
- Other (6) ________________________________________________
- I'm not sure, skip this question (7)

*Display This Question:*

*If Q13 = No*

Q13b Which of the following best explains why you have not downloaded the App?

- I haven't had a chance to download it (4)
- I couldn't figure out how to download it (5)
- I think it wouldn't work on my phone (7)
- I just didn't want to (8)
- Other (6) ________________________________________________
- I'm not sure, skip this question (9)

*Display This Question:*

*If Q13b = I just didn't want to*

Q13.b.i Why did you not want to download the App? (please select any/all that apply)

- Privacy concerns (4)
- I don't trust the HSE/government (5)
- I don't trust the technology companies (e.g. Google, Apple) (6)
- I don't think the App could be useful (7)
- Other (8) ________________________________________________
- ⊗I'm not sure, skip this question (9)

*Display This Question:*

*If Q11 != No*

Q14Do you have the App on your phone now?

- Yes (1)
- No I got a new phone and haven't downloaded it again yet (4)
- No I deleted it (2)
- No I never had it on my phone (3)

*Display This Question:*

*If Q14 = No I deleted it*

Q14.a Why did you delete the App? (please select any/all that apply)

- Battery related concerns (4)
- A friend or family member advised I delete it (5)
- Privacy concerns (6)
- Other (7) ________________________________________________
- ⊗I'm not sure, skip this question (8)

Q15 Please select the option below that best describes how you feel about the following statements (in relation to your most recent experience of the App)...

|  | Strongly Disagree (1) | Somewhat Disagree (2) | Neutral (3) | Somewhat Agree (4) | Strongly Agree (5) |
| --- | --- | --- | --- | --- | --- |
| **The App was easy to download and get started** (1) |  |  |  |  |  |
| **The App is easy to use** (2) |  |  |  |  |  |
| **The App looks professional and of high quality** (3) |  |  |  |  |  |
| **The App has slowed down my phone's performance** (4) |  |  |  |  |  |
| **The App causes my phone's battery to deplete quickly** (5) |  |  |  |  |  |

Q16 Is the main function of the HSE COVID Tracker App clear?

- Yes (1)
- No (2)
- I'm not sure (3)

*Display This Question:*

*If Q16!= No*

Q17.a So what do you feel the MAIN function of the App is?

- To help with Contact Tracing (4)
- To allow people to upload symptoms (5)
- To give people information on COVID-19 trends (6)
- I'm not sure, skip this question (7)

*Display This Question:*

*If Q16 = No*

Q17.b Why is the App's main function not clear? (please select any/all that apply)

- The HSE have not been clear about what its main function is (5)
- The App tries to do too much (4)
- Other (6) ________________________________________________
- ⊗I'm not sure, skip this question (7)

*Display This Question:*

*If Q13 = Yes*

Q18
The HSE COVID Tracker App has three distinct sections, each with a different function.
 
1. The HSE COVID Tracker App has a “Contact Tracing” feature which works in the background of your phone to anonymously record other phones with the App you come into contact with. For this feature to work your phone’s Bluetooth technology needs to be turned on. Have you had problems with your smartphone's Bluetooth technology and the App?

- Yes (1)
- No (2)
- Not applicable (4)

*Display This Question:*

*If Q18 = Yes*

Q18.a What problems did you have with your phone's Bluetooth and the COVID Tracker App?

________________________________________________________________

*Display This Question:*

*If Q13 = Yes*

2. Another section of the HSE COVID Tracker App allows you to inform the App if you have symptoms on any given day.

The next 2 questions focus on the "Check-In" section (screenshot shown below)...

*Display This Question:*

*If Q13 = Yes*

Q19 Over the past week, did you use the COVID Check-In function in the App?

- Everyday or nearly everyday (1)
- Several times (7)
- Once or twice (2)
- No (I didn't remember) (3)
- No (I wasn't aware of this function) (4)
- No (I don't want to use this function) (8)
- Not applicable (6)

*Display This Question:*

*If Q13 = Yes*

Q20 Do you think it is useful to be able to tell the App/the HSE if you are feeling unwell?

- Yes (1)
- No (2)
- I'm not sure (3)
- Not applicable (4)

*Display This Question:*

*If Q13 = Yes*

3. Finally, we would like to hear what you think of the "Updates" section of the App (screenshot shown below)...

This screen gives users the latest information relating to COVID-19 trends in Ireland.

*Display This Question:*

*If Q13 = Yes*

Q21 In August the HSE released a software update which allows the App to download the latest COVID-19 data relating to New Cases, Hospitalisations, ICU admissions and Deaths for the previous 2 weeks. Were you aware of this update?

 * note there was a further update of the App in September, making more language options available.

- Yes I am aware of the August update, I think I updated the Tracker to the latest version (1)
- Yes I am aware of the August update, but I don't think I have updated yet (2)
- No I wasn't aware of this update (3)
- Not applicable (9)

*Display This Question:*

*If Q13 = Yes*

Q22 Do you think it is useful to have the latest Irish COVID-19 data available on the App?

- Yes (1)
- No (2)
- I'm not sure (3)

*Display This Question:*

*If Q13 = Yes*

Q23 Have you received any notifications from the App about having been in close contact with a confirmed COVID-19 case?

- Yes (1)
- No (2)
- I'm not sure (4)

*Display This Question:*

*If Q23 = Yes*

Q23.a Were the instructions in the notification clear and easy to follow?

- Yes (1)
- No (2)

*Display This Question:*

*If Q23.a = No*

Q23.a.i Please explain why the instructions were not clear...

________________________________________________________________

Q24
**App Issues**
 Since the HSE COVID Tracker App was launched in early July, some users have experienced some technical difficulties.
 
When the App was launched, some people found out that their phones were too old to work with the App. This mainly affected older models of Android phones and the iPhone 6 and its older models.

- I was aware of this issue (1)
- I was not aware of this issue (2)

*Display This Question:*

*If Q24 = I was aware of this issue*

Q25 *"When the App was launched, some people found out that their phones were too old to work with the App. This mainly affected older models of Android phones and the iPhone 6 and its older models."*
 Please select the option that best applies to the above issue...

- This issue meant I could not download the App (1)
- This issue affected my confidence in the App a lot but I still downloaded it (3)
- This issue affected my confidence in the App a little but I still downloaded it (4)
- This issue did not affect my confidence in the App and I downloaded it (5)
- Not applicable (skip) (7)

Q26 When the App was launched, some people had to activate location services on their Android Phones for the COVID Tracker to be granted the necessary permissions to use the phone's bluetooth technology. It should be noted that this issue arises from Android software, not the COVID Tracker App (which does not record your location).

- I was aware of this issue (1)
- I was not aware of this issue (2)

*Display This Question:*

*If Q26 = I was aware of this issue*

Q27 *"When the App was launched, some people had to activate location services on their Android Phones for the COVID Tracker to be granted the necessary permissions to use the phone's bluetooth technology. It should be noted that this issue arises from Android software, not the COVID Tracker App (which does not record your location)."*
 Please select the option that best applies to the above issue...

- This issue led to me to not download the App (1)
- This issue led me to delete the App (permanently) (6)
- This issue led me to delete the App (temporarily) (2)
- This issue affected my confidence in the App a lot but I still downloaded it (3)
- This issue affected my confidence in the App a little but I still downloaded it (4)
- This issue did not affect my confidence in the App and I downloaded it (5)
- Not applicable (skip) (7)

Q28 In early August some people with the App noticed their battery was being used up very quickly and their phones became a lot hotter than usual. This was due to a problem with the Google Play Store and so only affected Android phones. It was fixed within a few days by a Google update.

- I was aware of this issue (1)
- I was not aware of this issue (2)

*Display This Question:*

*If Q28 = I was aware of this issue*

Q29 *"In early August some people with the App noticed their battery was being used up very quickly and their phones became a lot hotter than usual. This was due to a problem with the Google Play Store and so only affected Android phones. It was fixed within a few days by a Google update."*

 Please select the option that best applies to the above issue...

- This issue led to me to not download the App (1)
- This issue led me to delete the App (permanently) (6)
- This issue led me to delete the App (temporarily) (2)
- This issue affected my confidence in the App a lot but I kept it on my phone (3)
- This issue affected my confidence in the App a little but I kept it on my phone (4)
- This issue did not affect my confidence in the App and I kept it on my phone (5)
- Not applicable (skip) (7)

*Display This Question:*

*If Q13 = Yes*

Q30 Have you experienced any other problems with the HSE COVID Tracker App we haven't covered?

- Yes (1)
- No (2)

*Display This Question:*

*If Q30 = Yes*

Q30.a What other issue(s) did you have with the HSE COVID Tracker App?

________________________________________________________________

**Suggested App Changes**

Q31**.** Are there any changes to how the App **presents information** you would like to see? (please select any/all you think might be a good idea)

- I would like the App to give more up-to-date information on COVID-19 in Ireland (10)
- I would like the App to have more information about COVID-19 trends in my local area (11)
- I would like the App to tell me how many other people with the App I've been in close contact with recently (12)
- Other (please specify) (5) ________________________________________________
- ⊗No I don't think these ideas/changes would be useful (4)
- ⊗I'm not sure, skip this question (16)

Q32 Are there any changes to how the App **works** that you would like to see? (please select any/all you think might be a good idea)

- I would like the App to remind me to "check in" (14)
- I would like the App to buzz or notify me somehow if I was within 2m of another App user outside my household (1)
- I would like the App to buzz or notify me somehow if I was within 2m of another App user outside my household for more than 5-10 mins (3)
- It could be helpful if people could use the App as a "passport" to gain access to venues or events (2)
- The App should work with phones from other countries (i.e. tourists and other visitors) (6)
- The current age restriction of 18 years old should be removed (9)
- Other (please specify) (5) ________________________________________________
- ⊗No I don't think these ideas/changes would be useful (4)
- ⊗I'm not sure, skip this question (15)

Q33 Are there any changes to how **you enter data** into the App that you would like to see implemented? (please select any/all you think might be a good idea)

- I would allow the App to know my Age if it helped public health teams (1)
- I would allow the App to know my Ethnicity if it helped public health teams (2)
- I would allow the App to know my recent Location data if it helped public health teams (3)
- I would allow the App to know if I had been tested for COVID-19 recently if it helped public health teams (4)
- ⊗No I don't think these ideas/changes would be useful (5)
- ⊗I'm not sure, skip this question (6)

*Display This Question:*

*If Q13 != Yes*

Q13.a
You said above you have not yet downloaded the COVID Tracker App.
 
Is there any way it could be re-designed that would encourage you to download it?

- Yes, if it was modified (1)
- No, I'm not interested in downloading a contact tracing App (2)
- I'm not sure (3)

*Display This Question:*

*If Q13.a = Yes, if it was modified*

Q13.a.i What would you like to see changed? (please select any/all that apply)

- Get rid of the "extra" functionality and focus only on supporting Contact Tracing (4)
- A total redesign without the input of Google or Apple (5)
- Other (6) ________________________________________________
- ⊗I'm not sure, skip this question (7)

Q34
**App Usefulness**
  
In your opinion, is the COVID Tracker App helping our national response to COVID-19?

- Yes (4)
- No (5)
- I'm not sure (6)

*Display This Question:*

*If Q34 = Yes*

Q35.a Why do you think the COVID Tracker is helping our national response to COVID-19? (please select any/all that apply)

- It helps with contact tracing (4)
- It helps keep people informed on COVID-19 trends (5)
- It helps keep the HSE informed of peoples' symptoms (6)
- It helps with social solidarity / reminds us we're all in this together (9)
- Other (7) ________________________________________________
- ⊗Skip this question (8)

*Display This Question:*

*If Q34 = No*

Q35.b Why do you think the COVID Tracker is NOT helping our national response to COVID-19? (please select any/all that apply)

- Not enough people are using the App for it to be useful (7)
- I'm not convinced the technology involved in the App works (4)
- I haven't seen evidence that it is helping (10)
- Other (9) ________________________________________________
- ⊗Skip this question (8)

*Display This Question:*

*If Q34 = I'm not sure*

Q35.c Please tell us why you are unsure if the COVID Tracker is helping our national response to COVID-19... (please select any/all that apply)

- Not enough people are using the App for it to be useful (8)
- I'm not convinced the technology involved in the App works (9)
- I haven't seen evidence that it is helping (4)
- Other (6) ________________________________________________
- ⊗Skip this question (7)

Q36 **Follow-up Project**
 As part of this research work examining the COVID Tracker App we are looking to contact people to undertake a 20 minute telephone (or video call if you prefer) interview around the COVID Tracker App and what you think of it.
 
Please select Yes below if you would be interested to hear more about this further step in our research.

- Yes, I would like to participate (1)
- No thanks (2)

*Display This Question:*

*If Q36 = Yes, I would like to participate*

Q36.a
If you would like us to contact you, please enter your telephone number or email here.

Our research team will be in touch with you over the next 4 weeks.

You will of course be able to withdraw from the project at any time you choose...

________________________________________________________________
